# Poor Outcomes of Patients With NAFLD and Moderate Renal Dysfunction or Short-Term Dialysis Receiving a Liver Transplant Alone

**DOI:** 10.3389/ti.2022.10443

**Published:** 2022-12-09

**Authors:** Carlos Fernández-Carrillo, Yaming Li, Meritxell Ventura-Cots, Josepmaria Argemi, Dongling Dai, Ana Clemente-Sánchez, Andres Duarte-Rojo, Jaideep Behari, Swaytha Ganesh, Naudia L. Jonassaint, Amit D. Tevar, Christopher B. Hughes, Abhinav Humar, Michele Molinari, Douglas P. Landsittel, Ramon Bataller

**Affiliations:** ^1^ Center for Liver Diseases, Division of Gastroenterology, Hepatology and Nutrition, Department of Medicine, University of Pittsburgh Medical Center, Pittsburgh, PA, United States; ^2^ CIBERehd. Instituto de Salud Carlos III, Madrid, Spain; ^3^ Gastroenterología y Hepatología, IDIPHISA, Hospital Universitario Puerta de Hierro-Majadahonda, Madrid, Spain; ^4^ Department of Biomedical Informatics, University of Pittsburgh, Pittsburgh, PA, United States; ^5^ Thomas E. Starzl Transplant Institute, Department of Surgery, University of Pittsburgh Medical Center, Pittsburgh, PA, United States

**Keywords:** liver transplantation, non-alcoholic steatohepatitis, acute kidney injury, alcohol-related liver disease, chronic kidney disease

## Abstract

The outcomes of patients with moderate renal impairment and the impact of liver disease etiology on renal function recovery after liver transplant alone (LTA) are largely unknown. We explored whether NAFLD patients with pre-LTA moderate renal dysfunction (GFR 25–45 ml/min/1.73 m^2^) may be more susceptible to develop post-LTA severe renal dysfunction (GFR<15 ml/min/1.73 m^2^) than ALD patients, as well as other overall outcomes. Using the UNOS/OPTN database, we selected patients undergoing liver transplant for NAFLD or ALD (2006–2016), 15,103 of whom received LTA. NAFLD patients with moderate renal dysfunction were more likely to develop subsequent GFR<15 ml/min/1.73 m^2^ than ALD patients (11.1% vs. 7.38%, *p <* 0.001). Patients on short-term dialysis pre-LTA (≤12 weeks) were more likely to develop severe renal dysfunction (31.7% vs. 18.1%), especially in NAFLD patients, and were more likely to receive a further kidney transplant (15.3% vs. 3.7%) and had lower survival (48.6% vs. 50.4%) after LTA (*p* < 0.001 for all). NAFLD was an independent risk factor for post-LTA severe renal dysfunction (HR = 1.2, *p* = 0.02). NAFLD patients with moderate renal dysfunction and those receiving short-term dialysis prior to LTA are at a higher risk of developing subsequent severe renal dysfunction. Underlying etiology of liver disease may play a role in predicting development and progression of renal failure in patients receiving LTA.

## Graphical Abstract

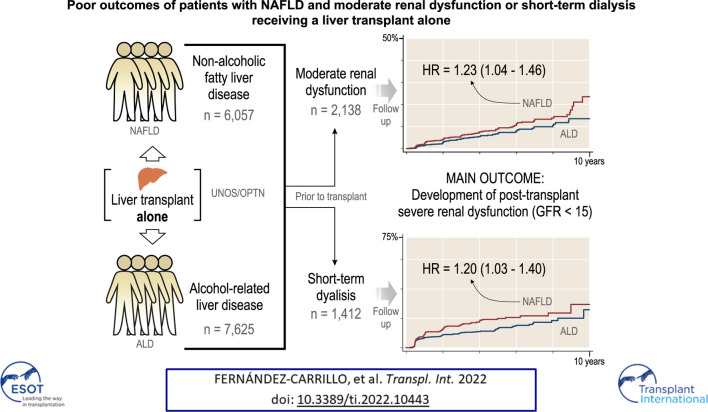

## Introduction

Non-alcoholic fatty liver disease (NAFLD) is a major health problem which has recently become the second leading indication for liver transplantation (LT) in the United States (1-4). NAFLD is also the most rapidly increasing indication for simultaneous liver-kidney transplant (SLKT) (5). In addition to a high prevalence of cardiovascular risk factors in NAFLD patients, there is an association between NAFLD and chronic kidney disease (CKD), which is independent of metabolic syndrome or cirrhosis (6-8). Moreover, a recent study has shown an independent association between pre-LT renal dysfunction and a worse graft and overall survival after transplant in NAFLD patients (9). In previous research we found that, compared with those with NAFLD, patients with alcohol-related liver disease (ALD) and renal dysfunction prior to LT have better outcomes after LT (10). This suggests that NAFLD may be more frequently associated with causes of renal dysfunction that have less reversion potential and that the etiology of liver disease may impact the recovery of renal function after LT. Previous studies are focused on patients with the most impaired renal function, such as those with creatinine (Cr) ≥ 2.5 mg/dl or with a need for dialysis (10,11). There is scarce information regarding outcomes of patients with moderate renal impairment after LT, and the impact of liver disease etiology on renal function recovery has not been fully addressed (12). Presumably, a higher incidence of structural kidney injury in the NAFLD population and overestimation of renal function when using serum Cr, may lead to overlook a significant and irreversible renal impairment in this vulnerable group of patients (13).

Beyond NAFLD-related indication, overall SLKT has been growing since 2002, when the Model for End-stage Liver Disease (MELD) score was adopted to guide graft allocation (14). The MELD score includes Cr. Renal dysfunction, which occurs in up to 30% of listed patients for LT, strongly influences the outcomes of patients with end-stage liver disease (15-21). The increase in SLKT has potentially resulted in important inequalities since kidney grafts may have been diverted from highly-prioritized kidney transplant (KT) candidates toward certain subsets of cirrhotic patients whose native kidneys might have recovered after liver transplant alone (LTA) (22-24). In view of this, certain proposals have been made by the Organ Procurement and Transplantation Network (OPTN) to offer some guidance on SLKT allocation, resulting in the inclusion of the latest consensus in OPTN official policies of 2017 (24-30). These are valuable criteria, but they still lack solid demonstration of a benefit in survival, and other studies show that glomerular filtration rate (GFR) alone may not guide SLKT indication (12,31,32). New predictive factors are needed in order to better support the decision making process. In this regard, it is remarkable that, with some exceptions, published studies overlooked a potential role of the etiology of liver disease for the indication of SLKT (5,9,10,12).

Based on these considerations, we hypothesize that NAFLD patients with renal dysfunction who receive LTA have worse kidney-related outcomes and reduced survival. Therefore, we aimed at exploring these variables in NAFLD patients with pre-LTA moderate renal dysfunction, compared to ALD patients with similar renal function impairment, who represent the other leading indication for LT. To better address the issue of SLKT indication, we assessed the same outcomes for those NAFLD patients on short-term dialysis vs. ALD patients.

## Patients and Methods

### Study Population

Using the United Network for Organ Sharing/Organ Procurement and Transplantation Network (UNOS/OPTN) database, we selected adult patients undergoing LT between January 1st, 2006 and January 1st, 2016 and with at least 1 year of available follow up data. This timeframe predates the UNOS SLKT policy (implemented in 2017) aimed at standardizing kidney allocation criteria in transplant candidates with acute or chronic kidney injury. Patients with only NAFLD or ALD as a single diagnosis were selected using codes 4214 and 4215 respectively, excluding any concomitant diagnoses. As previously described, we also considered NAFLD as the most likely underlying etiology of liver disease in those patients classified as cryptogenic or idiopathic cirrhosis (codes 4208 and 4213) and a body mass index (BMI) > 30 (3,5) In addition, diagnoses were manually reviewed where the code was 999 (“Other specify”), and patients matching the above criteria were included in the analysis. Patients with hepatocellular carcinoma or any other malignancy were excluded. Patients receiving both kidney and liver grafts on the same day or with a date mismatch of up to 24 h were classified as SLKT, whereas the rest of the patients were classified as recipients of LTA. Other multi-organ transplants were excluded. This study was approved by the University of Pittsburgh Institutional Revision Board as a consent-waived study with the number PRO18020615, and have therefore been performed in accordance with the ethical standards laid down in an appropriate version of the 2000 Declaration of Helsinki as well as the Declaration of Istanbul 2008.

### Variable and Outcome Definitions

Glomerular filtration rate (GFR) at the time of transplant is the standard parameter to assess kidney function endorsed by UNOS guidelines. GFR was estimated at that single time point by the formula 141 × min(Cr/κ, 1)^α^ × max(Cr/κ, 1)^−1.209^ × 0.993^Age^ × 1.018 [if female] × 1.159 [if black] (28,33). Clinically meaningful cutoffs for pre-LTA GFR were used to define three categories (>45, 45–25 and <25 ml/min/1.73 m^2^), of which the intermediate category (45–25 ml/min/1.73 m^2^) was defined as moderate renal dysfunction. An upper threshold of 45 ml/min/1.73 m^2^ is widely accepted as mildly to moderately decreased renal function (34). Although a lower cut-off of 30 ml/min/1.73 m^2^ is used in many studies for this category, 25 ml/min/1.73 m^2^ was used to cover a wider scope of clinical situations and follows UNOS/OPTN’s recommendations to define sustained acute kidney injury (AKI). The UNOS/OPTN database does not allow accurate distinction between acute or chronic kidney disease, while the OPTN policy recommends 25 instead of 30 for sustained acute kidney injury (AKI) (28,34). Given that Cr levels alone are commonly used in clinical practice, Cr at the time of transplant was also included in the analysis. Clinically meaningful cutoffs for pre-LTA Cr were used to define three categories of Cr elevation (<1.5 mg/dl, low; 1.5–2.5 mg/dl, moderate; > 2.5 mg/dl, high). Dialysis during the last week prior to LT is recorded in the UNOS/OPTN database and was used to define the group of patients on dialysis prior to LTA. Such patients were not included in the groups with pre-LTA GFR <25 or Cr > 2.5 mg/dl. Dialysis length was unavailable for LTA patients, for whom short-term dialysis (≤12 weeks) was assumed, since they did not receive a KT. Post-LTA severe renal dysfunction was an outcome defined as GFR <15 ml/min/1.73 m^2^ that persisted at least 6 months after LTA. This cut-off corresponds with the KDIGO G5 category and a Cr ≥ 4 mg/dl in patients within the age range of the study population. KT after LTA was matched with the LTA patients using patient code.

### Statistical Analysis

Summary statistics are reported as means (standard deviation) or n (%) for continuous or categorical variables, respectively. Wherever dispersion is high, median (interquartile range) is shown. The Chi-square test was used to analyze differences between categorical variables. A comparison of continuous variables between groups was performed using the Student t test. Survival rates were estimated using Kaplan-Meier curves of death-free, kidney transplant-free, and kidney failure-free survival and compared with the log-rank test. Cox proportional hazards and competing risk logistic models adjusted for age, gender, race, diabetes, and BMI (>40 vs. < 40) were developed to investigate which variables were independently associated with severe renal dysfunction and further kidney transplant after liver transplant alone. All reported *p*-values were two-tailed. The level of statistical significance was set at *p* < 0.05. Statistical analyses were performed with STATA software version 15.1.

## Results

**FIGURE 1:**
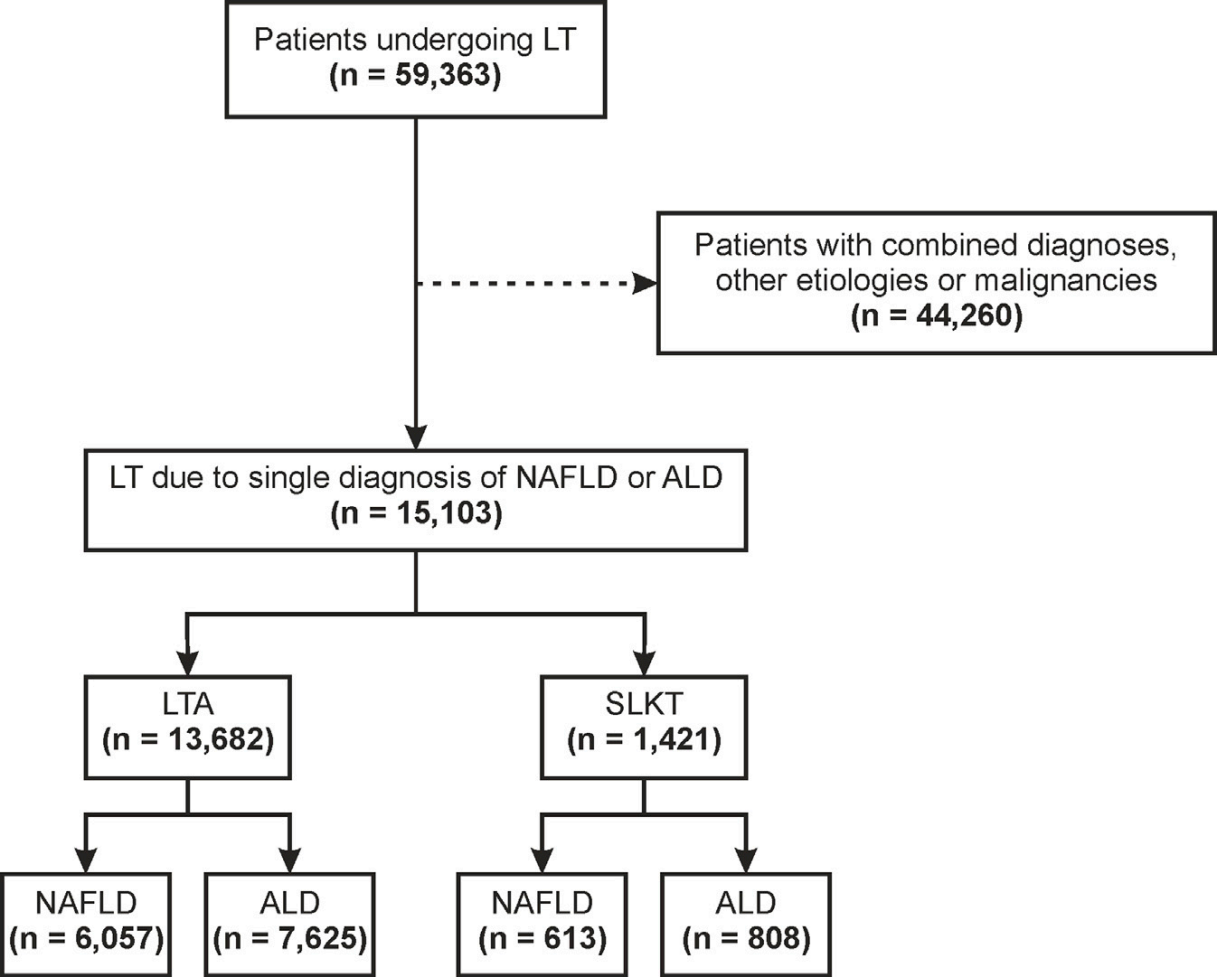
Flowchart for patient selection ALD, alcohol-related liver disease; LT, liver transplant; LTA, liver transplant alone; NAFLD, non-alcoholic fatty liver disease; SLKT, simultaneous liver-kidney transplant.

Between January 1st^,^ 2006 and January 1st^,^ 2016, we identified 59,363 patients that had received a LT across the United States. A total of 15,103 fulfilled the inclusion and exclusion criteria of the study and underwent LT because of NAFLD or ALD as the only indication (Figure 1). Of them, 13,682 (90.6%) underwent LTA and 1,421 (9.4%) underwent SLKT.

### Characteristics of Patients with NAFLD or ALD Without Pre-LTA Dialysis

A total of 12,088 patients out of 13,682 who underwent LTA (88.3%), did not receive dialysis treatment and had computable GFR. NAFLD was the indication for LTA in 5,427 (44.9%) of them while 6,661 (55.1%) underwent LTA for ALD. Within the group of NAFLD patients, there was a smaller predominance of male gender and a lower proportion of Hispanic and Black ethnicities as compared with ALD ones (male gender, 56.8% vs. 78.6%; Hispanic, 11.4% vs. 13.9%; Black, 1.9% vs. 3.7%; *p* < 0.001 for all) (Table 1). Additionally, NAFLD patients were older and had a higher BMI, as well as a higher proportion of type 2 diabetes mellitus (T2DM) (mean age, 59 vs. 55 years; mean BMI, 33 vs. 29; T2DM, 45.7% vs. 17.3%; *p* < 0.001 for all). Mean GFR was lower in NAFLD patients than in ALD patients (62.87 vs. 70.54 ml/min/1.73 m^2^, *p* < 0.001). ALD patients showed a slightly more impaired liver function with higher MELD scores (21 vs. 22; *p* < 0.001), due to higher bilirubin levels and INR.

**TABLE 1:** Baseline characteristics of LTA recipients, not receiving pre-transplant dialysis, according to the etiology of liver disease.

Characteristics	NAFLD	ALD	*p* value
	*n* = 5,427	*n* = 6,661	
Age (years)	59 ± 8	55 ± 9	**<0.001**
Gender (n, %)			**<0.001**
Male	3,080 (56.8)	5,237 (78.6)	
Female	2,347 (43.2)	1,424 (21.4)	
Race (n, %)			**<0.001**
White	4,560 (84)	5,319 (79.9)	
Hispanic	620 (11.4)	928 (13.9)	
Black	103 (1.9)	244 (3.7)	
Others	144 (2.7)	170 (2.5)	
BMI	33 ± 6	29 ± 5	**<0.001**
BMI > 40 (n, %)	549 (10.1)	191 (2.9)	**<0.001**
T2DM (n, %)	2,458 (45.7)	1,141 (17.3)	**<0.001**
GFR levels (ml/min/1.73 m^2^)	62.87 ± 29.5	70.54 ± 31.5	**<0.001**
GFR (n, %)			**<0.001**
GFR > 45 (n, %)	3,666 (68)	5,028 (76)	
GFR (25 – 45) (n, %)	1,216 (22)	1,064 (16)	
GFR < 25 (n, %)	545 (10)	569 (8.5)	
Creatinine levels (mg/dl)	1.41 ± 0.90	1.38 ± 0.92	0.085
Cr < 1.5 (n, %)	3,628 (66.9)	4,714 (70.7)	**<0.001**
Cr (1.5–2.5) (n, %)	1,330 (24.5)	1,337 (20.1)	**<0.001**
Cr > 2.5 (n, %)	469 (8.6)	610 (9.2)	**<0.001**
Albumin levels (g/dl)	3.01 ± 0.67	3.03 ± 0.68	0.13
Total Bilirubin levels (mg/dl)	3.6 (2–7.2)	4.8 (2.4–10.8)	**<0.001**
INR	1.87 ± 0.80	2.04 ± 1.59	**<0.001**
MELD score	21 ± 8	22 ± 9	**<0.001**
Ascites (n, %)	4,434 (82.2)	5,645 (85.3)	**<0.001**
SBP (n, %)	302 (5.7)	656 (10.0)	**<0.001**
On ventilator (n, %)	107 (2.0)	158 (2.4)	0.15
Portal vein thrombosis (n, %)	791 (14.7)	656 (9.9)	**<0.001**

*Others includes Asian, American Indian/Alaska Native, Hawaiian/other Pacific Islander, and Multiracial.

Values are shown as mean ± standard deviation, excepting bilirubin levels, which are shown as median (interquartile range) due to non-normal distribution.

ALD, alcohol-related liver disease; BMI, body mass index; Cr, serum creatinine; GFR, glomerular filtration rate; INR, international normalized ratio; LTA, liver transplant alone; NAFLD, non-alcoholic fatty liver disease; MELD, model for end-stage liver disease; SBP, spontaneous bacterial peritonitis; T2DM, type 2 diabetes mellitus.

### Impact of Moderate Renal Dysfunction Before LTA

**FIGURE 2:**
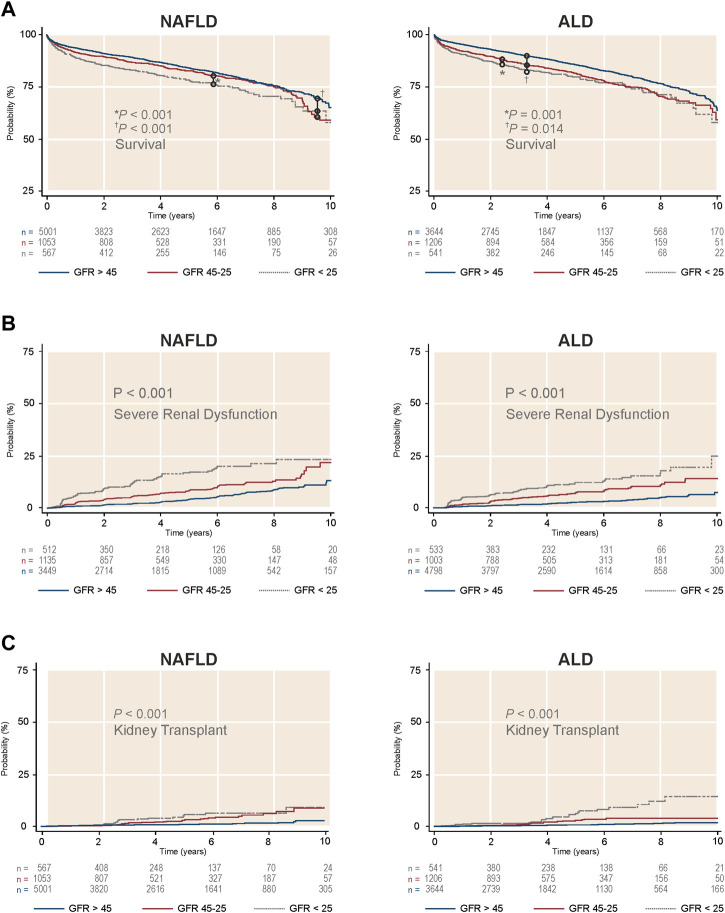
Survival and cumulative incidence of severe renal dysfunction and further kidney transplant in LTA recipients without prior dialysis according to GFR categories and stratified by etiology of liver disease. **(A)** Survival by liver disease etiology. **(B)** Cumulative incidence of severe renal dysfunction by liver disease etiology. **(C)** Cumulative incidence of kidney transplant indication by liver disease etiology. ALD, alcohol-related liver disease; GFR, glomerular, filtration rate; NAFLD, non-alcoholic liver disease.

**FIGURE 3:**
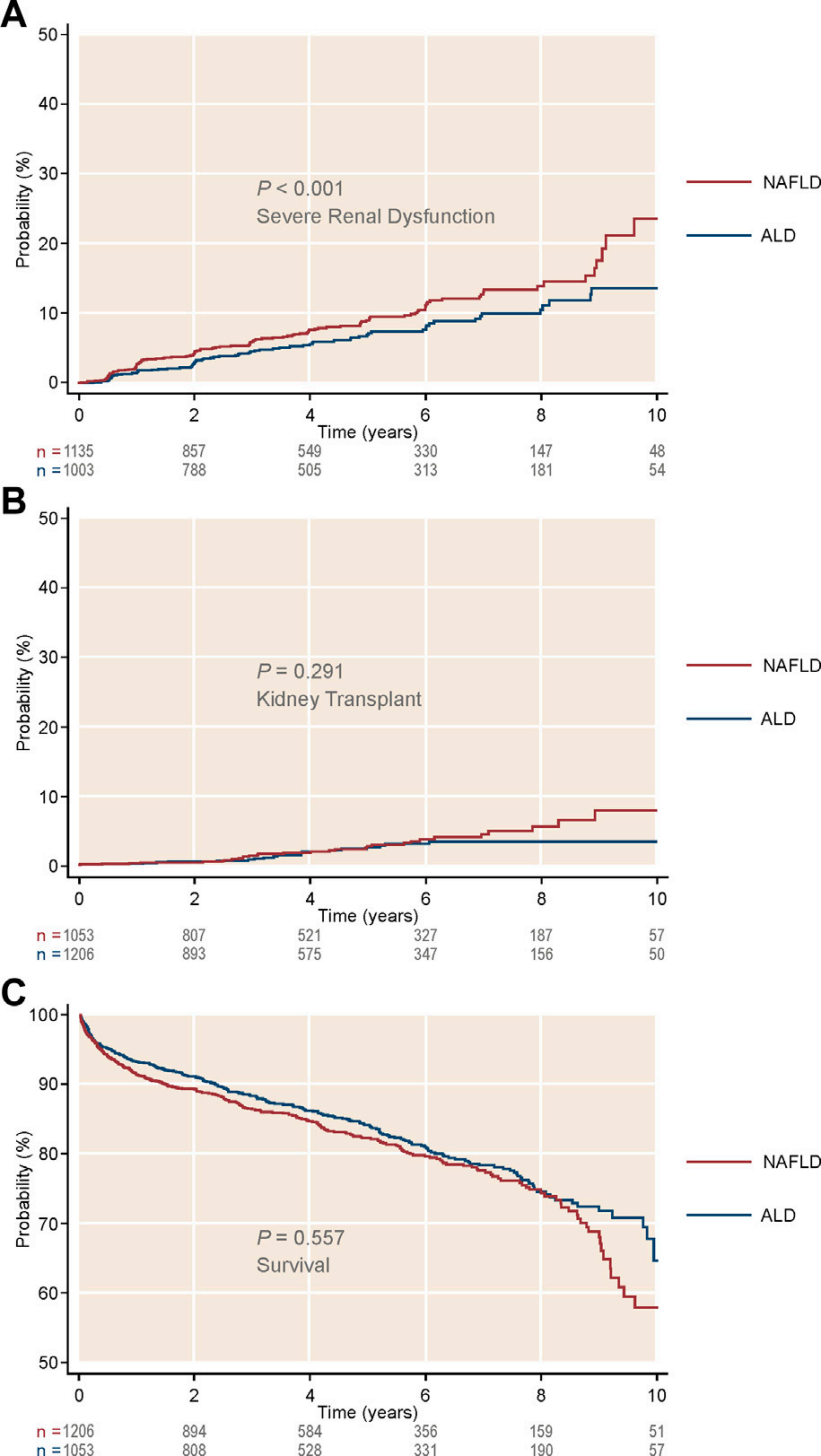
Cumulative incidence of kidney-related outcomes, as well as survival, in patients with intermediate glomerular filtration rate (45–25 ml/min/1.73 m^2^) after receiving a liver transplant alone. Stratification was done by liver disease etiology. **(A)** Cumulative incidence of severe renal dysfunction by etiology. **(B)** Cumulative incidence of kidney transplant by etiology. **(C)** Survival by etiology. ALD, alcohol-related liver disease; NAFLD, non-alcoholic liver disease.

First, we assessed the three pre-transplant GFR categories (>45, 45–25, and <25 ml/min1.73 m^2^) and their impact after LTA. Stratification of NAFLD or ALD patients by these categories showed three clearly differentiated curves for survival, development of post-LTA severe renal dysfunction (GFR <15 ml/min/1.73 m^2^) and further KT indication (*p* ≤ 0.01 for all) (Figure 2). Second, we focused on those patients with pre-transplant moderate renal impairment and compared them by liver-disease etiology, over a median time of 4.92 years (95% CI 4.80–4.99). Either according to predefined categories of GFR (45–25 ml/min1.73 m^2^) or Cr levels (1.5–2.5 mg/dl), NAFLD patients developed post-LTA severe renal dysfunction more frequently than ALD patients (GFR: 11.1% vs. 7.38%, *p* < 0.001; Cr: 10.5% vs. 7.1%, *p* = 0.045) (Figure 3; Supplementary Figure S1, respectively). In addition, NAFLD patients developed post-LTA severe renal dysfunction earlier than ALD patients, for whom this mainly happened after 2 years (Figure 2). There was no difference in overall post-transplant survival or need for KT between both etiologies in patients with moderate renal dysfunction, either using GFR or Cr levels (Figure 3; Supplementary Figure S1, respectively). However, of the patients with best renal function prior to LT (GFR >45 ml/min1.73 m^2^ or Cr levels <1.5 mg/dl), those with NAFLD still showed a higher cumulative incidence of post-LTA severe renal dysfunction vs. those with ALD (GFR: 5.22% vs. 3.23%, *p* = 0.006; Cr: 17.3% vs. 9.5%, *p* < 0.001) (Supplementary Figure S2).

Guided by the above unadjusted analysis, we built Cox proportional hazard models for incidence of severe renal dysfunction and for KT indication after LTA, in which the etiology of liver disease was included as an explanatory variable (Tables 2A,B). Both moderate or more severely impaired GFR prior to transplant were independent predictors of post-LTA severe renal dysfunction (GFR 45–25: HR 2.18, 95% CI 1.83–2.61; GFR <25: HR 3.61, 95% CI 2.99–4.36; *p* < 0.001 for both). These two categories were found to be as well the strongest risk factors impacting on further need of KT (GFR 45–25: HR 2.72, 95% CI 1.88–3.94; GFR <25: HR 4.77, 95% CI 3.26–7.00; *p* < 0.001 for both). Interestingly, NAFLD was an independent risk factor for development of post-LTA severe renal dysfunction (HR 1.23, 95% CI 1.04–1.46; *p* = 0.017), although it did not predict KT indication. In addition, Black race and T2DM, two well-known risk factors of CKD were also associated with severe renal dysfunction after LTA (Black race: HR 1.89, 95% CI 1.31–2.72, *p* = 0.001; T2DM: HR 1.74, 95% CI 1.47–2.07, *p* < 0.001). Likewise, T2DM was associated with KT indication after LTA (HR 1.71, 95% CI 1.20–2.44; *p* = 0.003). Given the high prevalence of T2DM within NAFLD patients, we assessed a potential interaction between etiology and T2DM, which was found not significant, suggesting that their impact may be independent (HR: 1.15, 95% CI 0.81–1.63). Age was independently associated with the need for KT only (HR 0.98, 95% CI 0.96–0.99), while gender or BMI >40 were not. Similar results were obtained using Cr levels categories instead of GFR (Supplementary Tables S1A, B). Finally, we performed a competing risk analysis for severe renal dysfunction, considering KT as the competing factor, which strongly supported Cox regression results (Supplementary Table S3).

**TABLE 2A:** Cox proportional hazards model for severe renal dysfunction development.

	HR	95% confidence interval	*p value*
NAFLD	1.231	1.037–1.462	**0.017**
Age	1.008	0.998–1.017	0.118
Gender (male)	0.975	0.830–1.46	0.763
Hispanic	1.075	0.863–1.340	0.517
Black	1.888	1.311–2.719	**0.001**
T2DM	1.744	1.471–2.067	**< 0.001**
BMI >40	0.968	0.718–1.304	0.829
GFR 45–25	2.184	1.829–2.609	**< 0.001**
GFR <25	3.608	2.989–4.356	**< 0.001**

BMI, body mass index; GFR, glomerular filtration rate; HR, hazard ratio; NAFLD, non-alcoholic fatty liver disease; T2DM, type 2 diabetes mellitus.

**TABLE 2B:** Cox proportional hazards model for kidney transplant after liver transplant alone in patients without pre-transplant dialysis.

	HR	95% confidence interval	*p* value
NAFLD	1.076	0.756–1.531	0.684
Age	0.980	0.962–0.998	**0.032**
Gender (male)	1.260	0.890–1.783	0.193
Hispanic	1.045	0.669–1.634	0.846
Black	1.123	0.458–2.756	0.800
T2DM	1.711	1.202–2.436	**0.003**
BMI >40	1.186	0.677–2.078	0.551
GFR 45–25	2.719	1.879–3.937	**< 0.001**
GFR <25	4.774	3.258–6.997	**< 0.001**

BMI, body mass index; GFR, glomerular filtration rate; HR, hazard ratio; NAFLD, non-alcoholic fatty liver disease; T2DM, type 2 diabetes mellitus.

### Analysis of Patients With Re-Transplantation After LTA

One hundred and sixty three patients out of 13,682 that underwent LTA (1.2%), had already received a previous liver transplant. Serum creatinine at the time of the second transplant was available in 130 patients. We performed a dedicated analysis to assess if this especial population showed similar outcomes to the ones of the overall LTA population. NAFLD was the indication in 59 (45.4%) of them while 71 (54.6%) underwent re-LTA for ALD (Supplementary Table S2). NAFLD patients were older, had a higher BMI and were more frequently affected by T2DM than ALD patients (mean age, 56 vs. 53, *p* = 0.024; mean BMI 31 vs. 27, *p* < 0.001; T2DM, 43% vs. 22%, *p* = 0.043). Baseline GFR and Cr levels did not differ between the two groups. However, among those with baseline moderate renal dysfunction, a total of 26.7% patients with NAFLD developed post-LTA severe renal dysfunction while such event was not observed in the ALD group (26.7% vs. 0%, *p* = 0.053) (Supplementary Figure S3A). Similar results were obtained when using the predefined moderate cutoff for Cr (moderate, 33.3% vs. 0%, *p* = 0.045) (Supplementary Figure S3B). Survival did not differ between both groups according to the etiology and no further KT indication did occur in this subgroup of patients.

### Impact of Pre-LTA Short-Term Dialysis According to the Etiology of Liver Disease

Short-term dialysis was performed in 1,576 patients (11.5%) out of 13,682 undergoing LTA prior to surgery. Within this population, 622 patients (39.5%) had NAFLD and 954 patients (60.5%) had ALD. MELD scores were significantly higher for ALD patients than for NAFLD patients (39 vs. 38, *p* < 0.001) (Table 3). Compared to LTA recipients that did not receive dialysis, these patients were younger and had a higher MELD score, mainly accounting for bilirubin levels (age, 54 vs. 57 years; MELD score, 38 vs. 22; bilirubin, 14.4 vs. 4.2 mg/dl; *p* < 0.001 for all), and exhibited ascites more frequently (93.9% vs. 83.9%, *p* < 0.001). Thus, the short-term dialysis group appeared to have a more severe clinical condition overall, related to either acute-on-chronic liver failure or advanced chronic liver disease.

**TABLE 3:** Baseline characteristics of the patients on short-term dialysis receiving a liver transplant alone, according to the etiology of liver disease.

Characteristic	NAFLD	ALD	*p* value
	*n* = 622	*n* = 954	
Age (years)	57 ± 9	52 ± 10	**<0.001**
Gender (n, %)			**<0.001**
Female	316 (50.8)	269 (28.2)	
Male	306 (49.2)	685 (71.8)	
Race			0.64
White	449 (72.2)	676 (70.9)	
Hispanic	136 (21.9)	212 (22.2)	
Black	16 (2.6)	38 (4.0)	
Others	21 (3.3)	28 (2.9)	
BMI	34 ± 6	29 ± 6	**<0.001**
BMI >40	94 (15.1)	58 (6.1)	**<0.001**
T2DM (n, %)	258 (42.0)	154 (16.4)	**<0.001**
Albumin levels (g/dl)	3.28 ± 0.78	3.28 ± 0.82	0.94
Total Bilirubin levels (mg/dl)	13.7 (5.7–29.1)	14.9 (6.9–28.8)	0.37
INR	2.42 ± 1	2.27 ± 1	**0.001**
MELD score	38 ± 6	39 ± 6	**<0.001**
Ascites (n, %)	581 (93.7)	893 (94.1)	0.15
SBP (n, %)	70 (11.4)	121 (13.0)	0.39
On ventilator (n, %)	133 (21.4)	249 (26.1)	**0.035**
Portal vein thrombosis (n, %)	99 (16.1)	82 (8.7)	**<0.001**

*Others includes Asian, American Indian/Alaska Native and Multiracial.

Values are shown as mean ± standard deviation, excepting bilirubin levels, which are shown as median (interquartile range) due to non-normal distribution.

ALD, alcohol-related liver disease; BMI, body mass index; INR, international normalized ratio; NAFLD, non-alcoholic fatty liver disease; MELD, model for end-stage liver disease; SBP, spontaneous bacterial peritonitis; T2DM, type 2 diabetes mellitus.

**FIGURE 4:**
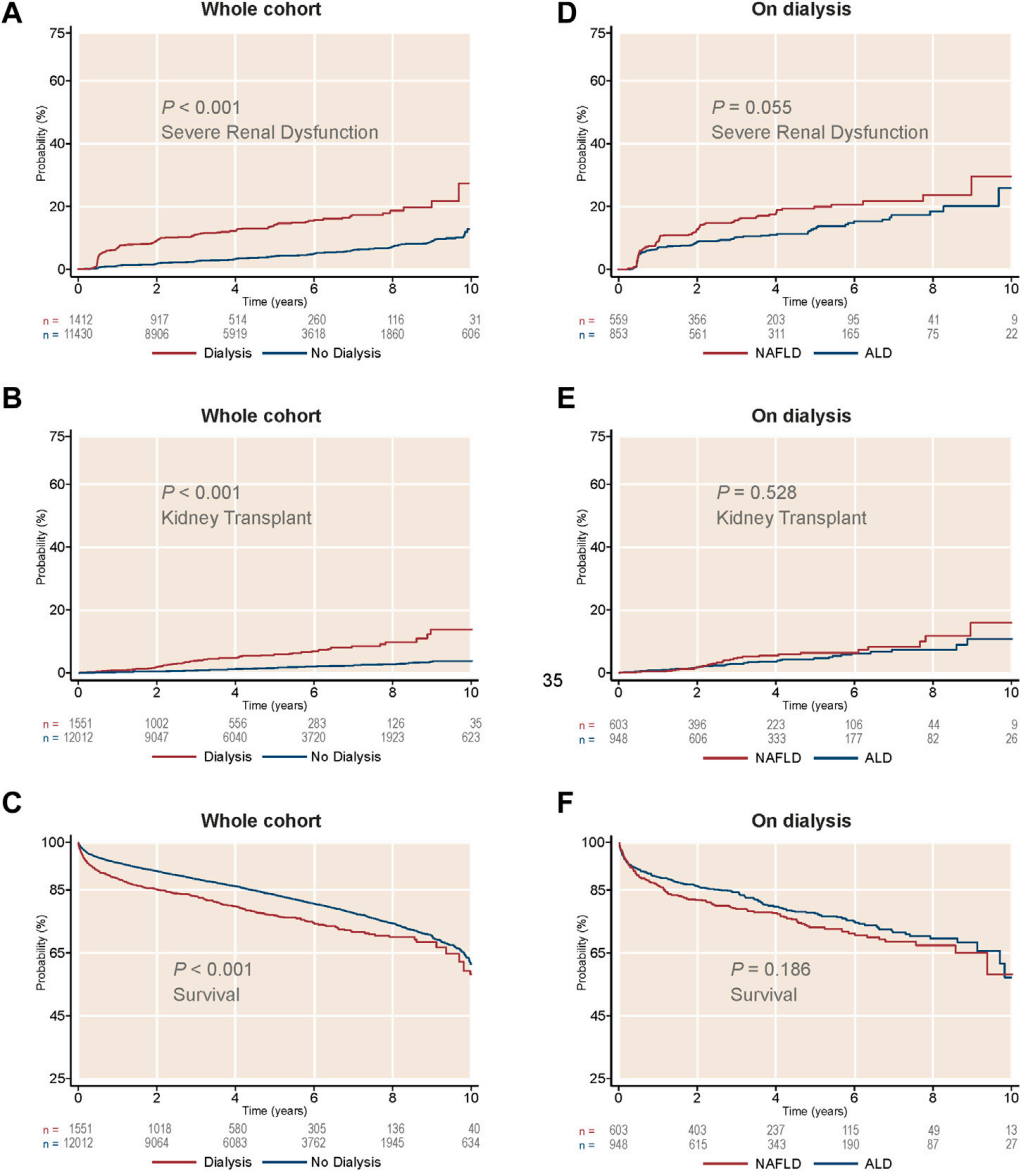
Cumulative incidence of kidney-related outcomes, as well as survival, in patients on short-term dialysis receiving a liver transplant alone. **(A)** Cumulative incidence of severe renal dysfunction by dialysis treatment. **(B)** Cumulative incidence of kidney transplant by dialysis treatment. **(C)** Survival by dialysis treatment. **(D)** Cumulative incidence of severe renal dysfunction in patients on dialysis, by liver disease etiology. **(E)** Cumulative incidence of kidney transplant in patients on dialysis, by liver disease etiology. **(F)** Survival in patients on dialysis, by liver disease etiology. ALD, alcohol-related liver disease; NAFLD, non-alcoholic liver disease.

After LTA, patients on prior short-term dialysis had a lower survival, developed severe renal dysfunction more frequently and were more likely to receive a further KT during a median follow-up of 3.98 years (95% CI 3.89–4.02) (survival, 48.64% vs. 50.4%; GFR <15 ml/min1.73 m^2^, 31.7% vs. 18.1%; KT, 15.3% vs. 3.7%; *p* < 0.001 for the three outcomes) (Figures 4A–C). When stratifying by etiology, patients with NAFLD on prior short-term dialysis showed a trend towards a greater frequency of post-LTA severe renal dysfunction (27.85% vs. 21.42%, *p* = 0.055) (Figure 4D). Cox proportional hazards models were constructed to explore the risk factors for severe renal dysfunction development after LTA in this population. Therefore, pre-LTA GFR was substituted by the binary covariate prior short-term dialysis. Interestingly, NAFLD etiology was an independent risk factor for post-LTA severe renal dysfunction (HR 1.20, 95% CI 1.03–1.40; *p* = 0.020), yet prior dialysis was the risk factor that showed the strongest impact (HR 3.29, 95% CI 2.79–3.89; *p* < 0.001) (Table 4). Age, male gender, Black race, and T2DM were other factors independently associated with this outcome (Age: HR 1.01, 95% CI 1.001–1.02; male gender: HR 1.33, 95% CI 1.14–1.56; T2DM: HR 1.71, 95% CI 1.46–2.00; *p* < 0.05 for all). Again, we did not find significant interaction between etiology and T2DM (HR: 1.08, 95% CI 0.79–1.47).

**TABLE 4:** Cox proportional hazards model for severe renal dysfunction after liver transplant alone, including those receiving short-term dialysis prior to transplant.

	HR	95% confidence interval	*p* value
NAFLD	1.201	1.029–1.402	0.020
Age	1.009	1.001–1.018	0.033
Gender (male)	1.335	1.140–1.562	<0.001
Hispanic	1.037	0.854–1.259	0.713
Black	2.092	1.521–2.877	<0.001
T2DM	1.709	1.462–1.998	<0.001
BMI >40	1.163	0.903–1.496	0.242
Dialysis	3.290	2.786–3.886	<0.001

BMI, body mass index; HR, hazard ratio; NAFLD, non-alcoholic fatty liver disease; T2DM, type 2 diabetes.

## Discussion

NAFLD is a major cause of advanced liver disease in the United States and worldwide, and is an increasing indication for LT and SLKT (3,4,35). The number of SLKT has been rising during the MELD era due to frequent kidney dysfunction related to chronic liver disease. On the other hand, NAFLD has been independently associated with CKD (6-8). However, the impact of the underlying etiology of the liver disease has been largely disregarded in previous studies on LTA and SLKT. A recent study showed suboptimal post-LT outcomes in patients with NAFLD and renal dysfunction (GFR <30 ml/min1.73 m^2^) including LTA and SLKT (9). Whether the underlying etiology influences the outcome of renal dysfunction after LT remains elusive. Therefore, we aimed at addressing this knowledge gap. In the current study, we show that the impact of mild or moderate renal dysfunction was more pronounced in patients with NAFLD than in ALD patients.

After stratification of patients receiving LTA into three clinically relevant categories based on GFR or Cr, we identified three respective groups who had different rates of survival, development of severe renal dysfunction (GFR <15 ml/min1.73 m^2^), and need for KT. When focusing on moderate renal dysfunction before transplantation (45–25 ml/min/1.73 m^2^), NAFLD patients showed increased incidence of post-LTA severe renal dysfunction compared to patients with ALD. This is clinically relevant since mild to moderate Cr elevation is commonly found in NAFLD patients listed for liver transplantation. The ability to predict renal function recovery after LT in patients with chronic liver disease is quite limited, and may potentially be more difficult in patients with some degree of structural kidney injury, which is common in NAFLD (6-8,36,37). Even among those patients with good pre-LTA renal function, NAFLD patients developed post-LTA severe renal dysfunction more frequently, which strongly suggests the existence of underlying structural kidney disease with poor functional recovery potential. The lack of kidney function recovery was also observed in NAFLD patients undergoing liver re-transplantation, which reinforces this notion. Prospective studies looking for serum biomarkers predictive of renal function recovery in patients with moderate renal dysfunction listed for LTA are warranted.

Our multivariable models confirmed that liver disease etiology is an independent risk factor for developing severe renal dysfunction after LTA, which was 23% more likely in patients with NAFLD. Renal function prior to LTA estimated by GFR or Cr levels was also found to be an independent risk factor in determining development of severe renal dysfunction and need for KT after LTA. Other independent risk factors for marked renal dysfunction after LTA were T2DM and Black race. T2DM, which was also a risk factor for receiving a kidney transplant during follow-up, is a well-known cardiovascular risk factor involved in metabolic syndrome and CKD. Particularly, NAFLD patients have a high incidence of T2DM (38), which in our cohort accounted for 45.7% compared to 17.3% in ALD patients. Black patients are particularly predisposed to developing CKD (39). Although this association may be mediated through a higher prevalence of arterial hypertension, we could not assess this factor in the UNOS/OPTN database. Disregarding race, arterial hypertension may be a potential confounder that could not be controlled. The fact that Black race was more frequent within ALD patients points at T2DM and potentially NAFLD itself, as main factors for the development of severe renal dysfunction. Even though metabolic syndrome is intrinsically associated with CKD, BMI >40 was not found to have an independent association in our models. All these findings suggest that there may be some subclinical underlying kidney damage in patients with NAFLD (6-8). A convoluted crosstalk among liver, visceral adipose tissue inflammation and kidneys, in addition to cardiovascular risk factors, may account for this structural renal injury (40,41).

Regarding patients who received dialysis before LT, it is important to conceptually differentiate CKD with long-term dialysis from short-term dialysis due to AKI mainly attributed to liver disease (e.g., hepatorenal syndrome or acute tubular necrosis). Concerning the latter, the required duration of dialysis to consider SLKT has been a matter of debate. The existing evidence is based on retrospective single-center experiences, spanning from 4 to 12 weeks, with significant variations among centers (22,25,26,42). Moreover, the precise indications and timing for dialysis in liver patients is not well defined, with significant heterogeneity in clinical practice (27). In our study, patients with NAFLD on short-term dialysis showed a clear trend to develop more frequently severe renal dysfunction after LTA. The multivariable analysis again showed NAFLD etiology as an independent risk factor for this outcome, along with other known risk factors such as age, male gender, Black race, and T2DM. The latest OPTN proposals and policies, issued after our study period, are fairly conservative and recommend 6 weeks of dialysis length in order to consider SLKT (24,27,28). Although this recommendation is expected to improve outcomes, new studies are needed to address whether the etiology of liver disease may be incorporated in the decision-making process.

The retrospective nature of our study limited our ability to adjust for confounding factors. While UNOS/OPTN database offers a large and representative sample over the US, some specific data were lacking, and the influence of potential changes in clinical practice over a ten-year span may not be properly reflected. Particularly, detailed history on calcineurin inhibitor use is lacking, which may influence kidney-related outcomes. Moreover, Cr level, which is known to be suboptimal for renal risk stratification in this setting, was the only marker available to estimate renal function. To mitigate this issue, we estimated GFR, which is the OPTN standard, by using the most accurate equation to date. Cr-based GFR may still be suboptimal since GFR equations were developed in non-cirrhotic patients and overestimate renal function in this population, yet this is an issue in real clinical practice rather than a study limitation (43). In addition, we could not discern between CKD and AKI, or the type of AKI, both critical conditions to guide clinical management and potential indication for SLKT (24,28,44). In this regard, AKI and CKD are closely related and AKI precedes transition to CKD in approximately 20% of patients. In the opposite direction, CKD is also a strong predictor of AKI (45,46). Given the increasing evidence that NAFLD patients have some degree of CKD, the previous considerations may be applied to this specific population. Finally, the lack of data on the precise duration of dialysis in patients receiving LTA is a limitation of the study. Assuming that most centers followed the well-accepted UNOS criteria, it is plausible that patients with indication for dialysis who underwent subsequent LTA were on renal replacement therapy for a short period. Prospective studies are needed including the precise indication and duration of dialysis prior to transplant.

In conclusion, our study shows that the underlying etiology of liver disease (NAFLD vs. ALD, the two leading LT indications) may play a role in predicting the development and progression of renal failure in patients receiving LTA. In addition, even if short-term dialysis before LTA has a strong impact on kidney-related outcomes regardless of the etiology of liver disease, it seems to be more pronounced in patients with NAFLD. Our results support the hypothesis that NAFLD patients have some degree of structural kidney disease, which could negatively impact the renal function recovery after LTA. Prospective studies are required to identify predictors and biomarkers of renal function recovery after LTA.

## Data Availability

Publicly available datasets for all OPTN Member transplant centers were analyzed in this study. Formal export requests can be made here: https://optn.transplant.hrsa.gov/data/view-data-reports/request-data/ United Network for Organ Sharing/Organ Procurement and Transplantation Network (UNOS/OPTN) database.

## Supplementary Material

Supplementary Figure S1 Cumulative incidence of kidney-related outcomes, as well as survival, in patients with moderate renal dysfunction (creatinine 1.5–2.5 mg/dl) after receiving a liver transplant alone. Stratification was done by liver disease etiology. **(A)** Cumulative incidence of severe renal dysfunction by etiology. **(B)** Cumulative incidence of kidney transplant by etiology. **(C)** Survival by etiology. ALD, alcohol-related liver disease; NAFLD, non-alcoholic liver disease.

Supplementary Figure S2 Cumulative incidence of severe renal dysfunction in patients with glomerular filtration rate >45 ml/min/1.73 m^2^ or low serum creatinine levels (<1.5 mg/dl) after receiving a liver transplant alone. Stratification was done by liver disease etiology. **(A)** Cumulative incidence of severe renal dysfunction in patients with glomerular filtration rate >45 ml/min/1.73 m^2^. **(B)** Cumulative incidence of severe renal dysfunction in patients with creatinine <1.5 mg/dl. ALD, alcohol-related liver disease; GFR, glomerular filtration rate; NAFLD, non-alcoholic fatty liver disease.

Supplementary Figure S3 Cumulative incidence of severe renal dysfunction after receiving a second liver transplant alone, in patients with moderate renal dysfunction. Stratification was done by liver disease etiology. **(A)** Cumulative incidence of severe renal dysfunction after receiving a second liver transplant alone in patients with glomerular filtration rate 45–25 ml/min/1.73 m^2^. **(B)** Cumulative incidence of severe renal dysfunction after receiving a second liver transplant alone in patients with serum creatinine levels 1.5–2.5 mg/dl. ALD, alcohol-related liver disease; GFR, glomerular filtration rate; NAFLD, non-alcoholic liver disease.

## Abbreviations

AKI, acute kidney injury; ALD, alcohol-related liver disease; BMI, body mass index; CKD, chronic kidney disease; CI, confidence interval; Cr, serum creatinine; GFR, glomerular filtration rate; HR, hazard ratio; INR, international normalized ratio; KDIGO, Kidney Disease Improving Global Outcomes; KT, kidney transplant; MELD, Model for End-Stage Liver Disease; NAFLD, non-alcoholic fatty liver disease; LT, liver transplantation; LTA, liver transplant alone; OPTN, Organ Procurement and Transplantation Network; SLKT, simultaneous liver-kidney transplantation; T2DM, type 2 diabetes mellitus; UNOS, United Network for Organ Sharing.
