# Supplementary material for: Poor Outcomes of Patients With NAFLD and Moderate Renal Dysfunction or Short-Term Dialysis Receiving a Liver Transplant Alone
Source: Transpl Int. 2022 Dec 9;35:10443. doi: 10.3389/ti.2022.10443 (PMC9784907; doi:10.3389/ti.2022.10443)
Supplement: Supplementary file 1 [file Table1.docx]

**Supporting Table 1.** Cox proportional hazards models for severe renal dysfunction development and kidney transplant after liver transplant alone in patients without pre-transplant dialysis. Renal function covariate estimated by serum creatinine.

**Supporting Table 1A.** Cox proportional hazards model for severe renal dysfunction development. Renal function covariate estimated by serum creatinine.

|  | **HR** |  | **95% Confidence Interval** | | ***P* value** |
| --- | --- | --- | --- | --- | --- |
| NAFLD | 1.175 |  | 0.984-1.403 |  | 0.075 |
| Age | 1.005 |  | 0.995-1.015 |  | 0.334 |
| Gender (male) | 1.293 |  | 1.027-1.472 |  | **0.024** |
| Hispanic | 1.095 |  | 0.870-1.380 |  | 0.440 |
| Black | 2.091 |  | 1.456-3.003 |  | **< 0.001** |
| T2DM | 1.794 |  | 1.501-2.145 |  | **< 0.001** |
| BMI > 40 | 1.089 |  | 0.804-1.476 |  | 0.581 |
| *Cr (1.5 – 2.5) | 2.232 |  | 1.863-2.672 |  | **< 0.001** |
| *Cr > 2.5 | 4.352 |  | 3.557-5.325 |  | **< 0.001** |

Abbreviations: BMI, body mass index; Cr, serum creatinine; HR, hazard ratio; NAFLD, non-alcoholic fatty liver disease; T2DM, type 2 diabetes mellitus.

*(mg/dL)

**Supporting Table 1B.** Cox proportional hazard model for kidney transplant. Renal function covariate estimated by serum creatinine.

|  | **HR** |  | **95% Confidence Interval** | | ***P value*** |
| --- | --- | --- | --- | --- | --- |
| NAFLD | 1.065 |  | 0.748 | 1.515 | 0.727 |
| Age | 0.986 |  | 0.968 | 1.005 | 0.143 |
| Gender (male) | 0.999 |  | 0.709 | 1.411 | 1.000 |
| Hispanic | 1.056 |  | 0.675 | 1.652 | 0.811 |
| Black | 1.047 |  | 0.427 | 2.572 | 0.919 |
| T2DM | 1.791 |  | 1.257 | 2.551 | **0.001** |
| BMI > 40 | 1.189 |  | 0.678 | 2.085 | 0.546 |
| *Cr (1.5 – 2.5) | 3.072 |  | 2.144 | 4.401 | **< 0.001** |
| *Cr > 2.5 | 6.107 |  | 4.129 | 9.033 | **< 0.001** |

Abbreviations: BMI, body mass index; Cr, serum creatinine; HR, hazard ratio; NAFLD, non-alcoholic fatty liver disease; T2DM, type 2 diabetes mellitus.

*(mg/dL)
