# Supplementary figures and images for: Poor Outcomes of Patients With NAFLD and Moderate Renal Dysfunction or Short-Term Dialysis Receiving a Liver Transplant Alone
Source: Transpl Int. 2022 Dec 9;35:10443. doi: 10.3389/ti.2022.10443 (PMC9784907; doi:10.3389/ti.2022.10443)

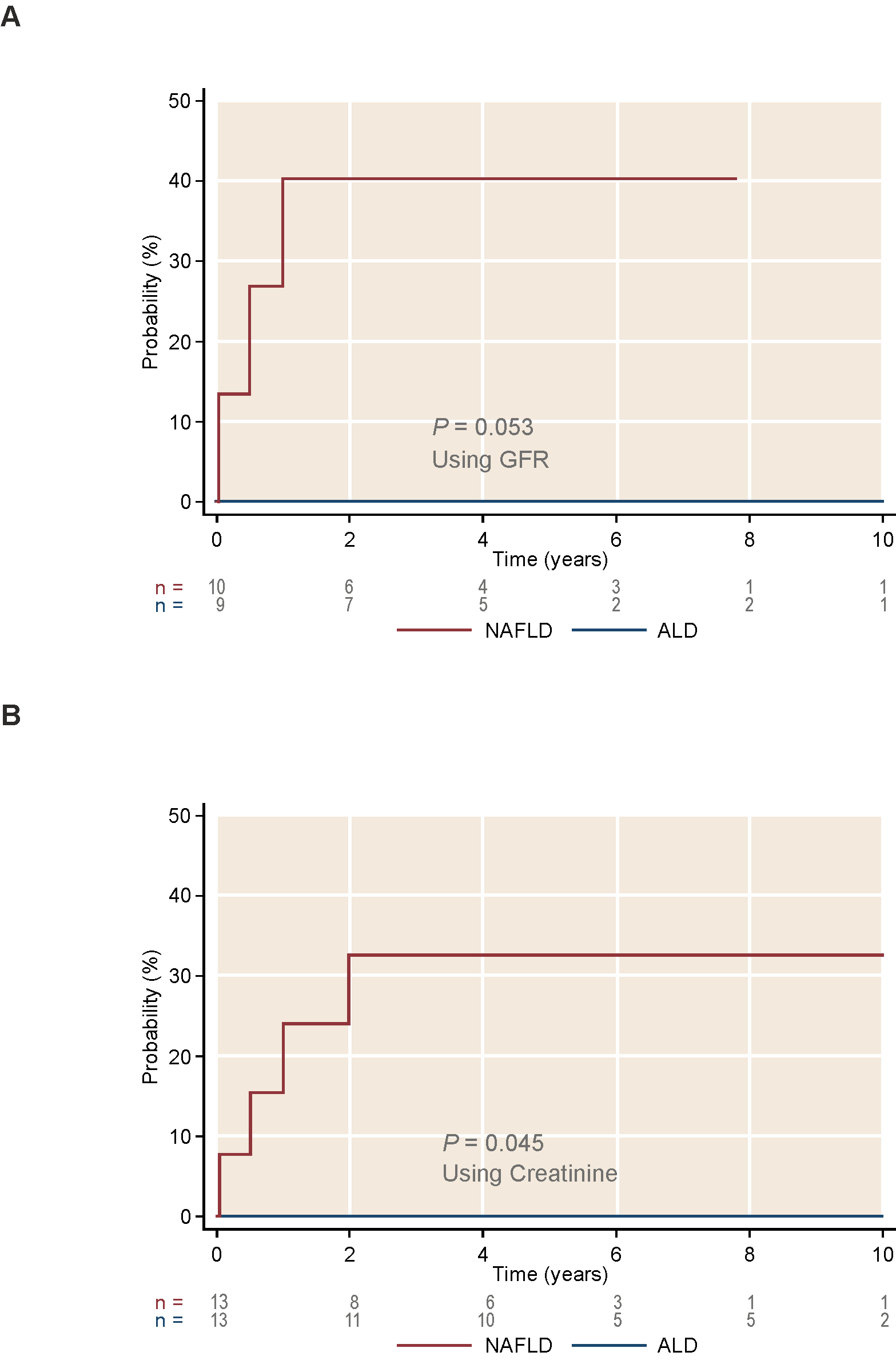

Supplement: Supplementary file 2 [file Image3.TIF]

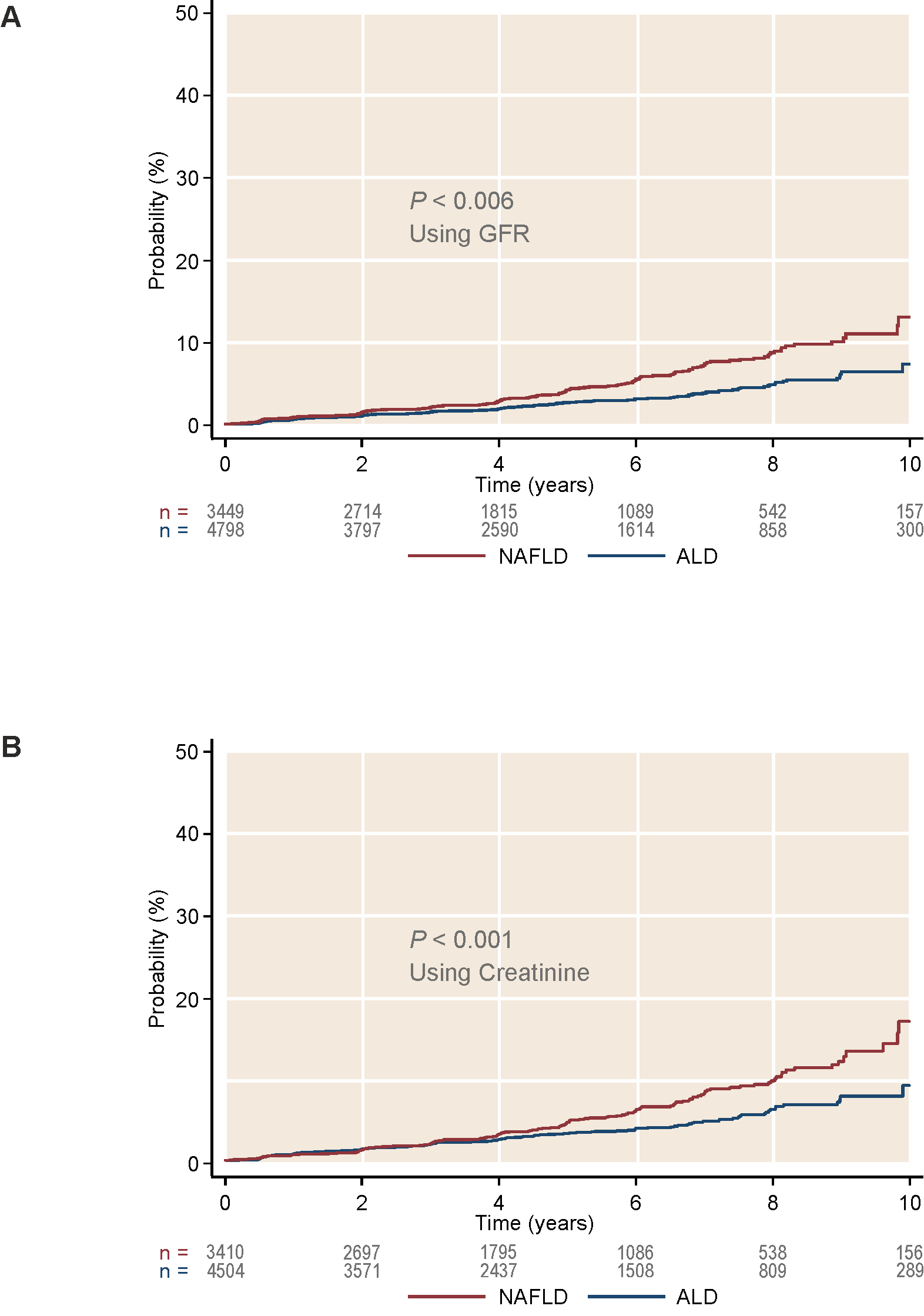

Supplement: Supplementary file 3 [file Image2.tif]

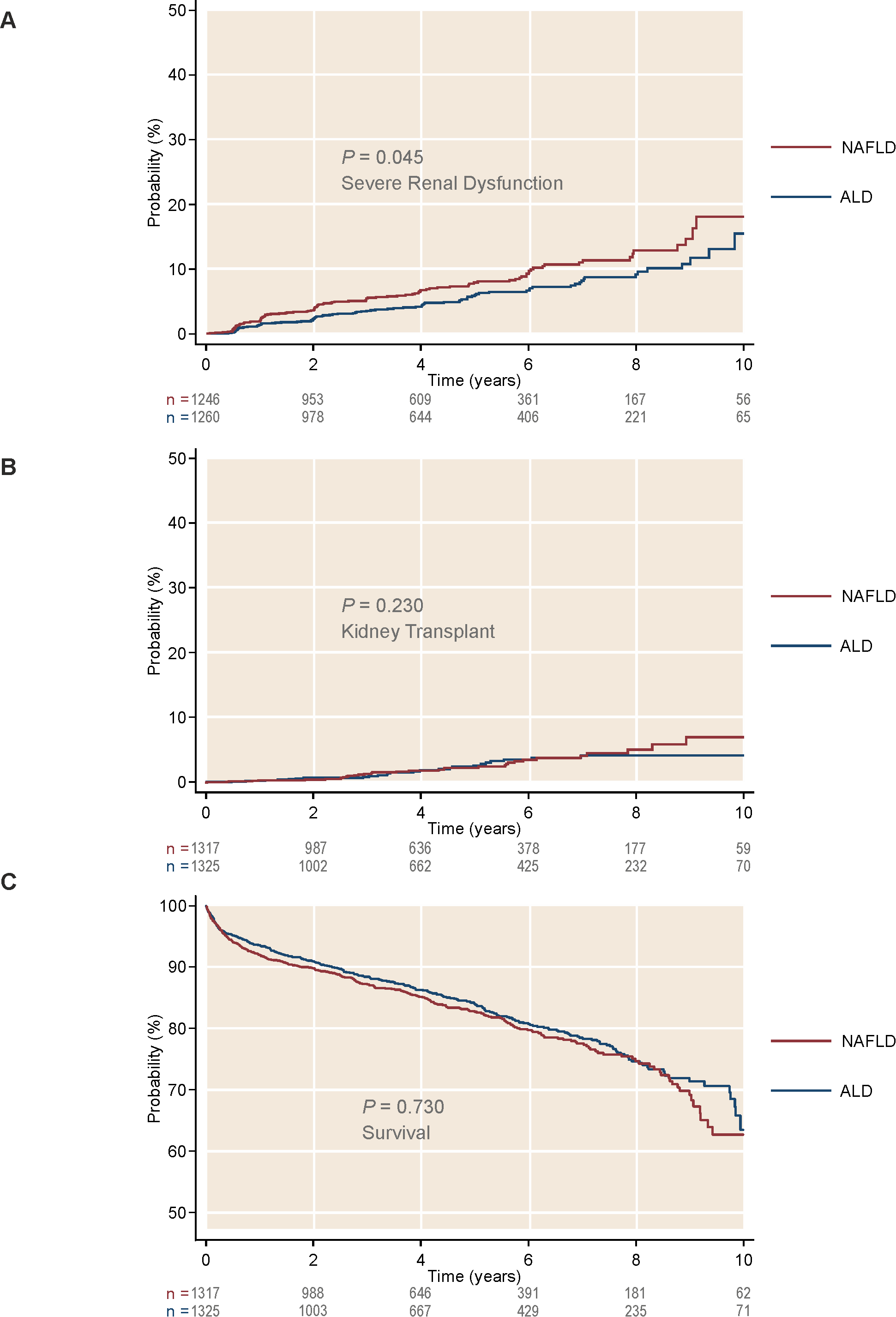

Supplement: Supplementary file 4 [file Image1.tif]
