# Supplementary material for: Poor Outcomes of Patients With NAFLD and Moderate Renal Dysfunction or Short-Term Dialysis Receiving a Liver Transplant Alone
Source: Transpl Int. 2022 Dec 9;35:10443. doi: 10.3389/ti.2022.10443 (PMC9784907; doi:10.3389/ti.2022.10443)
Supplement: Supplementary file 5 [file Table2.docx]

**Supporting Table 2.** Baseline characteristics of patients with LTA retransplant without prior dialysis.

|  | **NAFLD** | **ALD** | ***P* value** |
| --- | --- | --- | --- |
| **Characteristics** | **n = 59** | **n = 71** |  |
| Age (years) | 56 ± 8 | 53 ± 9 | **0.024** |
| Gender (n, %) |  |  | 0.35 |
| Male | 37 (62.7) | 51 (71.8) |  |
| Female | 22 (37.3) | 20 (28.2) |  |
| Race (n, %) |  |  | **0.047** |
| White | 46 (78) | 61 (86) |  |
| Hispanic | 9 (15) | 7 (9.9) |  |
| Black | 0 (0) | 3 (4.1) |  |
| Others | 4 (7) | 0 (0) |  |
| BMI | 31 ± 6 | 27 ± 5 | **<0.001** |
| BMI > 40 (n, %) | 4 (6.8) | 0 (0) | **0.040** |
| T2DM (n, %) | 25 (43.1) | 15 (21.7) | **0.043** |
| GFR levels (mL/min/1.73m2) | 59.89 ± 33.5 | 69.18 ± 37 | 0.140 |
| GFR (n, %) |  |  | 0.37 |
| GFR > 45 (n, %) | 35 (59) | 49 (69) |  |
| GFR (25 – 45) (n, %) | 15 (25) | 11 (15) |  |
| GFR < 25 (n, %) | 9 (15) | 11 (15) |  |
| Creatinine levels (mg/dL) | 1.62 ± 1.04 | 1.47 ± 1.00 | 0.41 |
| Cr (n, %) |  |  | 0.58 |
| Cr < 1.5 (n, %) | 33 (55.9) | 46 (64.8) |  |
| Cr (1.5 – 2.5) (n, %) | 16 (27.1) | 15 (21.1) |  |
| Cr > 2.5 (n, %) | 10 (17.0) | 10 (14.1) |  |
| Albumin levels (g/dL) | 2.67 ± 0.69 | 2.76 ± 0.75 | 0.50 |
| Total Bilirubin levels (mg/dL) | 15.86 ± 14.84 | 13.52 ± 14.07 | 0.36 |
| INR | 1.84 ± 1 | 1.81 ± 1 | 0.87 |
| MELD score | 24 ± 9 | 22 ± 10 | 0.22 |
| Ascites (n, %) | 43 (72.9) | 49 (71.0) | 0.21 |
| SBP (n, %) | 7 (11.9) | 2 (3.1) | 0.084 |
| On ventilator (n, %) | 12 (20.3) | 12 (16.9) | 0.65 |
| Portal vein thrombosis (n, %) | 10 (17.0) | 12 (17.1) | 1.00 |

*Others includes Asian and American Indian/Alaska Native.

ALD, alcohol-related liver disease; BMI, body mass index; Cr, serum creatinine; INR, international normalized ratio; GFR, glomerular filtration rate; NAFLD, non-alcoholic fatty liver disease; MELD, model for end-stage liver disease; SBP, spontaneous bacterial peritonitis; T2DM, type 2 diabetes mellitus.
