# Supplementary material for: Poor Outcomes of Patients With NAFLD and Moderate Renal Dysfunction or Short-Term Dialysis Receiving a Liver Transplant Alone
Source: Transpl Int. 2022 Dec 9;35:10443. doi: 10.3389/ti.2022.10443 (PMC9784907; doi:10.3389/ti.2022.10443)
Supplement: Supplementary file 6 [file Table3.docx]

**Supporting Table 3.** Competing risk regression for time to develop severe renal dysfunction after LTA in patients without pre-transplant dialysis, considering kidney transplant as a competing factor.

|  | **SHR** | **95% Confidence Interval** | ***P value*** |
| --- | --- | --- | --- |
| NAFLD | 1.270 | 1.055-1.531 | **0.012** |
| Age | 1.012 | 1.001-1.023 | **0.032** |
| Gender (male) | 0.976 | 0.815-1.169 | 0.790 |
| Hispanic | 1.066 | 0.835-1.363 | 0.605 |
| Black | 2.154 | 1.448-3.205 | **< 0.001** |
| T2DM | 1.711 | 1.421-2.060 | **< 0.001** |
| BMI > 40 | 0.942 | 0.672-1.320 | 0.727 |
| GFR 45-25 | 2.086 | 1.718-2.533 | **< 0.001** |
| GFR < 25 | 3.573 | 2.876-4.440 | **< 0.001** |

BMI, body mass index; GFR, glomerular filtration rate; SHR, subdistribution hazard ratio; NAFLD, non-alcoholic fatty liver disease; T2DM, type 2 diabetes mellitus.
