# Supplementary material for: Poor Outcomes of Patients With NAFLD and Moderate Renal Dysfunction or Short-Term Dialysis Receiving a Liver Transplant Alone
Source: Transpl Int. 2022 Dec 9;35:10443. doi: 10.3389/ti.2022.10443 (PMC9784907; doi:10.3389/ti.2022.10443)
Supplement: Supplementary file 7 [file Table4.docx]

**Supporting Table 4.** Outcomes for patients receiving a liver transplant alone with pre-transplant moderate renal dysfunction (glomerular filtration rate 45-25 mL/min/1.73m^2^), as well as for those with pre-transplant dialysis.

|  | GFR 45-25^1^ | |  | On dialysis | |  |
| --- | --- | --- | --- | --- | --- | --- |
|  | NAFLD | ALD | *P* value | NAFLD | ALD | *P* value |
| Survival, n (%) | 915 (75.25) | 805 (75.66) | 0.557 | 444 (71.38) | 732 (76.73) | 0.186 |
| Severe renal dysfunction, n (%)^2^ | 126 (11.1) | 74 (7.38) | **< 0.001** | 93 (16.64) | 103 (12.08) | 0.055 |
| Kidney transplant, n (%) | 30 (2.47) | 20 (1.88) | 0.291 | 31 (4.98) | 42 (4.4) | 0.528 |

^1^mL/min/1.73m^2^

^2^Defined as GFR < 15 mL/min/1.73m^2^ at least 6 months after liver transplant alone.

ALD, alcohol-related liver disease; GFR, glomerular filtration rate; NAFLD, non-alcoholic fatty liver disease
